# TasTY: Development and evaluation of a taste test for quantifying taste sensitivity among preschool children—test–retest reliability, application, and comparison between children with and without prior SARS-CoV-2 infection

**DOI:** 10.1007/s00431-026-07534-w

**Published:** 2026-10-19

**Authors:** Johannes Weißenfeld, Christoph Maier, Christine H. Meyer-Frießem, Anna Teresa Hoffmann, Thomas Lücke, Anne Schlegtendal

**Affiliations:** 1 University Children’s Hospital, St. Josef Hospital, Ruhr University Bochum, Bochum, Germany; 2 Department of Anaesthesiology, Intensive Care and Pain Medicine, KLW St. Paulus GmbH St. Marien Hospital Lünen, Lünen, Germany

**Keywords:** Taste test, Preschool children, Gustatory function, Test–retest reliability, SARS-CoV-2, Taste sensitivity

## Abstract

Taste dysfunction gained attention during the coronavirus disease 2019 (COVID-19) pandemic; however, validated objective tests for preschool children remain limited. We aimed to develop a feasible, reliable test quantifying taste sensitivity among children aged 3–6 years and to compare performance between children with and without prior SARS-CoV-2 infection. We included 68 symptom-free children (median age 5.1 years; interquartile range (IQR) 3.9–5.9): 36 without known SARS-CoV-2 infection (control group, CG) and 32 with confirmed prior infection (SARS-CoV-2 infection group, C19-G), tested 79 days after infection (median; IQR 56–112). Children completed a self-developed Taste Test for Young Children (TasTY), a double-blind, playful two-alternative forced-choice test with 10 samples in randomized order (sweet, sour, and salty at three concentrations each; water served as a control). Twenty controls were retested a median of 62 days later (IQR 38–64). Outcomes were total score (0–10), feasibility, task comprehension, and test–retest reliability, assessed using the intraclass correlation coefficient (ICC), standard error of measurement (SEM), and smallest real difference (SRD). Feasibility was high (3% dropout), and 89.4% correctly classified water. Total score test–retest reliability was good (ICC = 0.73; pooled SEM = 0.76; SRD = 2.11). Total scores were similar between CG and C19-G (8.37 ± 1.5 vs. 8.19 ± 1.5; *p* = 0.63). Descriptively, three-year-olds (*n* = 17) had lower, more variable scores.

*Conclusion*: TasTY provides an age-appropriate, reliable, objective method for quantifying taste sensitivity among preschoolers, supporting standardized group comparisons and longitudinal assessment.

**Table:** 

**What is Known:** • *COVID-19 increased awareness of taste dysfunction; however, evidence among young children relies mainly on surveys and parental reports.* • *Objective taste tests for preschool children remain limited.*
**What is New:** • *TasTY is a feasible, standardized, and objective test for quantifying taste sensitivity among children aged 3–6 years and goes beyond parental and subjective reports.* • *Its standardized 0–10 score showed good test–retest reliability and may support group comparisons and longitudinal assessment of preschool children with taste-related conditions.*

**Supplementary Information:**

The online version contains supplementary material available at https://doi.org/10.1007/s00431-026-07534-w.

## Introduction

Taste is important for food acceptance/avoidance, nutrition, and growth; even minor undetected impairments may affect development. Early taste experiences also shape later eating habits and dietary patterns, with long-term health implications [1–3]. Taste and smell dysfunctions became highly visible during the coronavirus disease 2019 (COVID-19) pandemic. Severe acute respiratory syndrome coronavirus 2 (SARS-CoV-2) infection has been associated with olfactory and gustatory dysfunctions among adults [4, 5] and children [6, 7], which may persist beyond acute illness [6]. Taste dysfunctions were more frequent during the Delta era but declined in later pandemic phases [8–10]. During the Omicron era, hypogeusia no longer appeared pathognomonic for SARS-CoV-2 infection [11].

Despite reports of olfactory and gustatory dysfunction among pediatric populations [12, 13], data on prevalence, severity, and recovery among children aged 3–6 years remain limited [14]. Objective, age-appropriate taste tests are still rare [14, 15], and studies rely largely on parental reports and surveys, which correlate poorly with objective measures of smell or taste and are not well suited for identifying sensory disturbances [16–20]. This leaves a gap in objective taste assessment in preschoolers. Validated psychophysical methods for older children and adults include the three-drop method and the Taste Strip Test [21–23]; the latter is brief, practical, reproducible, and validated in children aged ≥ 6 years [18]. However, these methods require verbal identification of taste qualities, which is difficult for younger children [15, 18].

Therefore, we developed a taste test for children aged 3–6 years to quantify taste sensitivity, assess feasibility and test–retest reliability, and compare performance among children with and without prior SARS-CoV-2 infection.

## Patients and methods

### Study design and ethical approval

We conducted a prospective cross-sectional study at the University Children’s Hospital, Ruhr University Bochum, Germany, from April to December 2022, including children aged 3–6 years. The study was approved by the Ethics Committee of Ruhr University Bochum (24 March 2022; 20–6927) and registered at the German Clinical Trials Register (DRKS00030718). Written informed consent was obtained from guardians.

### Participant eligibility and group assignment

Children aged 3–6 years were recruited via kindergartens, flyers, and the University Children’s Hospital. The exclusion criteria were acute infection potentially affecting taste perception within 3 days before testing, severe infection requiring hospitalization, or ear-nose-throat (ENT) surgery within the preceding 14 days. Children with a positive SARS-CoV-2 antigen or PCR test within the previous 4 months were assigned to the prior SARS-CoV-2 infection group (C19-G); those without known positive testing or suspected household infection formed the control group (CG). Children with household exposure without confirmed infection were excluded due to high transmission risk [24]. For test–retest reliability, CG participants were invited for repeat testing; 20 were retested, whereas the others did not respond or became ineligible after interim SARS-CoV-2 infection. This subgroup constituted the retest group (RG).

### Test administration and procedure

Tests were conducted in a familiar environment (home or kindergarten), in a quiet, separate room at a familiar room temperature, between 9 a.m. and 5 p.m. All TasTY assessments were conducted by the same researcher (JW), with at least one parent or childcare worker present. Children fasted for at least 60 min before testing; water was allowed. Guardians completed a structured questionnaire on demographics, medical history, SARS-CoV-2 course, and ENT-related history or symptoms, including prior ENT surgery, recurrent ENT infections, mouth breathing, snoring, and symptoms suggestive of obstructive sleep apnea, as potential covariates influencing sensory perception. TasTY (**Tas**te **T**est for **Y**oung Children) is a brief, child-adapted two-alternative forced-choice (2AFC) assessment developed by our study team. To enhance motivation and attention, children helped a mother dinosaur find a “magic potion” to cure her child based on prior narrative-based approaches [25–29] (Supplementary Text S1 in Online Resource). Children received 10 coded samples in randomized order: sweet, sour, and salty in three concentrations each, plus a water control. Umami and bitter were excluded primarily to limit test duration and participant burden and maintain feasibility in preschool-aged children; limited umami familiarity and expected bitter aversiveness were secondary considerations [30, 31]. For each sample, the children classified the solution as flavored (“magic potion”) or unflavored (“water”) by placing the cup on one of two marked fields on a playing board (Supplementary Fig. S1). Children rinsed with water between samples to minimize carryover; an initial sip familiarized them with the unflavored reference. One point was awarded per correct choice, yielding a total score ranging from 0 to 10; the ideal result would be 9 magic potions and one cup of water. Response times from ingestion to decision were summed as cumulative response time to assess whether task familiarity reduced retest response time and whether speed was associated with total score.

Both the child and the tester were blinded to taste quality and concentration. Cups were coded on the underside beforehand and set aside in the order selected by the child to ensure a blinded evaluation.

### Taste stimuli and concentrations

Testing was guided by German Institute for Standardization/International Organization for Standardization (DIN ISO) 3972 sensory standards [32]. The stimuli used were sucrose, citric acid, and sodium chloride. Locally sourced tap water was used as solvent, control and for mouth rinsing between samples; calculated mean total mineralization was 317.6 mg/L (range 286.8–346.1 mg/L) based on reports from the supplying waterworks in 2022 (water analysis reports are available upon request). Distilled water was not used because aversive reactions had occurred during the development phase of the test. For each taste quality, three concentrations were used: 24, 36, and 60 mmol/L sucrose; 1.3, 2.0, and 3.1 mmol/L citric acid; and 15.4, 21.4, and 27.4 mmol/L sodium chloride. The concentrations were selected to create three difficulty levels intended to correspond to detection rates of approximately 50%, 75%, and > 90%. Levels were guided by published pediatric detection thresholds for sweet and salty stimuli [25, 27] and by DIN ISO 3972 standards for sour stimuli [32]. Water served as a comprehension control [33]. Solutions were freshly prepared in sterilized glass bottles, coded, and administered at room temperature.

### Outcomes and statistical analysis

The primary outcome was taste sensitivity assessed by the TasTY total score (0–10). Secondary outcomes were feasibility (dropout rate), task comprehension (recognition of water as a control), and test–retest reliability for the total score in the RG. Associations of total score with age, sex, and cumulative response time were examined; within the C19-G, days since infection were additionally assessed. Fatigue was explored by comparing the error rates across the 10 sample positions.

Detection rates by taste quality, age and concentration were calculated descriptively (Supplementary Table S1). Normal distribution was assessed using the Kolmogorov‒Smirnov and Shapiro‒Wilk tests. Nonnormally distributed variables are generally reported as medians (interquartile ranges, IQRs). Because of ceiling effects in the bounded 0–10 score [34], the score and cumulative response time are additionally reported as means ± standard deviations (SDs) with bootstrapped 95% confidence intervals to facilitate interpretation and comparisons across groups and time points. Categorical variables were compared using χ^2^ tests and group differences using Mann‒Whitney U or Kruskal‒Wallis tests. Age differences were first assessed across four age groups using the Kruskal–Wallis test; an exploratory Mann–Whitney U test then compared three-year-olds with children aged 4–6 years, with Bonferroni adjustment for both analyses. Paired test‒retest comparisons were performed using Wilcoxon signed-rank tests. Correlations were assessed with Spearman’s ρ. Multiple linear regression was used to examine the combined effects of age, sex, and group (CG/C19-G) on the total score.

Test–retest reliability was quantified using the intraclass correlation coefficient (ICC; two-way mixed-effects model, single measures, absolute agreement), interpreted according to Cicchetti [35]. Because the ICC reflects relative reliability, absolute measurement precision was additionally assessed using the standard error of measurement (SEM = SD × √(1 − ICC)) based on the pooled test–retest standard deviation (SD) [36–38]. The smallest real difference (SRD = 1.96 × √2 × SEM) estimated the minimum within-person change unlikely to reflect measurement error [37]. Bland‒Altman plots assessed agreement and potential systematic bias between the test and retest results by plotting individual differences (y-axis) against the mean of both measurements (x-axis) and indicating the 95% limits of agreement (LoA; mean difference ± 1.96 SD); narrower intervals indicate better agreement [39]. Analyses and figure preparation were performed using IBM SPSS Statistics (version 29.2) and Microsoft Excel; two-sided p < 0.05 was considered to indicate statistical significance.

## Results

**Fig. 1:**
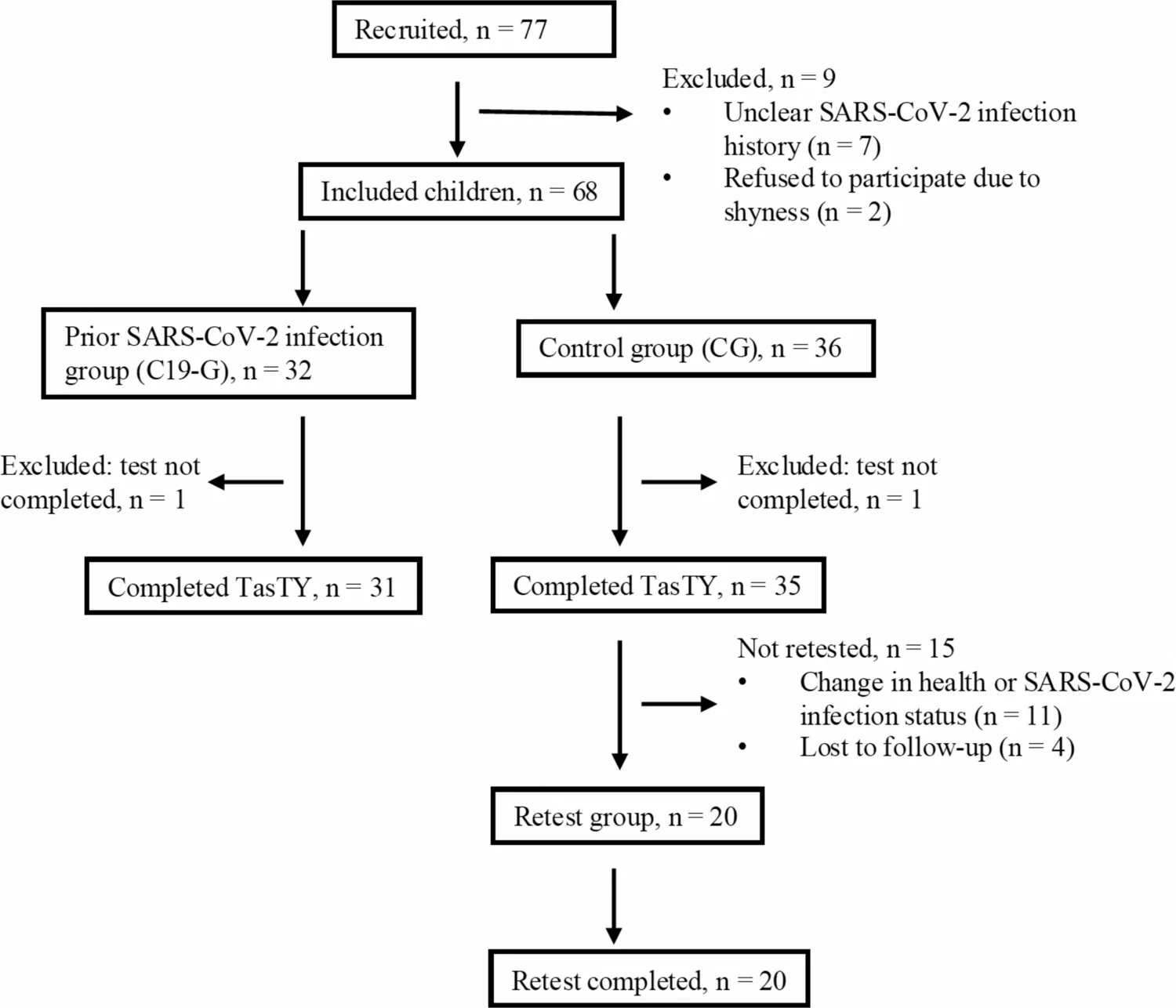
Flowchart of participant recruitment, group allocation, and retest inclusion

Seventy-seven children were recruited between April and December 2022. After exclusions due to unclear SARS-CoV-2 infection history (*n* = 7) and shyness (*n* = 2), 68 children were included (CG: *n* = 36; C19-G: *n* = 32) (Fig. 1). Each group had one dropout; both groups were included descriptively but excluded from the score analyses, leaving 66 children for score analysis (CG: *n* = 35; C19-G: *n* = 31).

In the C19-G, testing occurred a median of 79 days postinfection (IQR 56–112); 28/32 (87.5%) reported symptomatic courses, and all were symptom-free at testing. In the CG, 20 children completed retesting after a median of 62 days (IQR 38–64). Demographics were comparable between groups except for more frequent ENT-related complaints in the C19-G (Table 1). Previous ENT surgery, where present, had occurred a median of 130.5 days before taste testing (IQR 84.5–290 days).

**Table 1:** Demographic and clinical characteristics of the study population

	Control group, CG (*n* = 36)	Retest group, RG (*n* = 20)	SARS-CoV-2 infection group, C19-G (*n* = 32)
Age (years), median, interquartile range (IQR)	5.0 (3.9–5.9)	4.8 (4.1–6.0)	5.1 (4.5–5.9)
3 years	12 (33.3%)	5 (25.0%)	6 (18.8%)
4 years	6 (16.7%)	6 (30.0%)	9 (28.1%)
5 years	10 (27.8%)	4 (20.0%)	9 (28.1%)
6 years	8 (22.2%)	5 (25.0%)	8 (25.0%)
Females	18 (50.0%)	10 (50.0%)	15 (46.9%)
ENT-associated complaints^a^	2 (5.6%)	1 (5.0%)	10 (31.3%)^*^
Previous ENT surgery^b^	3 (8.3%)	1 (5.0%)	5 (15.6%)
History of allergic rhinitis	0 (0.0%)	0 (0.0%)	2 (6.3%)

The study cohort comprised a control group (CG) and children with prior SARS-CoV-2 infection (C19-G); the retest group (RG) was a subset of the CG. Values are n (%) unless stated otherwise; age is presented as the median (interquartile range, IQR). ENT, ear-nose-throat. ^a^ENT-associated complaints included recurrent otitis media/effusions, snoring, predominant mouth breathing, obstructive sleep apnea, speech delay, clumsy speech, and frequent upper respiratory tract infections. ^b^Previous ENT surgeries included polypectomy/adenoidectomy, tympanostomy tube insertion, and tonsillectomy. ^*^p < 0.05 versus the CG (χ^2^ test)

## Primary outcome: taste sensitivity

### Total score, group comparisons, and associations

The total scores did not differ between groups (CG: 8.37 ± 1.50 vs. C19-G: 8.19 ± 1.50; Mann–Whitney U, *p* = 0.63). Excluding children who misclassified the control solution (7/66) did not materially change the results (CG: 8.47 ± 1.44 vs. C19-G: 8.30 ± 1.52; *p* = 0.79). Scores did not differ significantly among the four age groups (Kruskal–Wallis test, *p* = 0.10). Descriptively, three-year-olds had lower and more variable scores than older children (Table 2 and Supplementary Fig. S2). Exploratory analysis showed lower scores in three-year-olds than in children aged 4–6 years combined (Mann–Whitney U = 258.5, Z =  − 2.38, nominal *p* = 0.017, Bonferroni-adjusted *p* = 0.034; r = 0.29). The regression model used to examine the effects of age, sex, and group on the total score was not significant (F(3,62) = 2.21; *p* = 0.096; R^2^ = 0.097) and no predictor was independently significant (age: B = 0.30, 95% CI − 0.03 to 0.64, *p* = 0.077; female sex: B = 0.51, 95% CI − 0.22 to 1.23, *p* = 0.165; CG versus C19-G: B = 0.19, 95% CI − 0.52 to 0.91, *p* = 0.594). The cumulative response time showed no discernible age-related pattern (Table 2). Spearman analyses revealed an association between age and total score in the CG (ρ = 0.34; *p* = 0.048) but not in the C19-G. No associations were found between total score and cumulative response time or, within the C19-G, days since infection.

**Table 2:** TasTY total score and cumulative response time by age and group

Age, years	n (CG)	Score (CG)	Time (CG), s	n (C19-G)	Score (C19-G)	Time (C19-G), s
3	11	7.45 (6.4–8.6)	55.9 (44.4–68.6)	6	7.50 (6.0–9.0)	45.1 (38.8–51.7)
4	6	9.00 (8.5–9.5)	51.2 (39.2–65.0)	9	8.56 (7.9–9.2)	40.4 (36.1–44.9)
5	10	8.40 (7.5–9.2)	45.9 (39.7–52.5)	9	8.33 (7.4–9.1)	46.0 (37.4–56.9)
6	8	9.13 (8.5–9.6)	44.8 (39.8–49.8)	7	8.14 (7.4–8.9)	64.8 (37.3–100.7)

Values are means with bootstrapped 95% confidence intervals (CIs) for the TasTY total score (0–10) and cumulative response time, stratified by age in the control group (CG) and among children with prior SARS-CoV-2 infection (C19-G). The cumulative response time denotes the sum of the response times from ingestion to the decision across all 10 samples, in seconds. n indicates the number of children per age group

## Stimulus-level detection rates

The detection rates increased with concentration across all three taste qualities and were broadly comparable between the CG and the C19-G. Overall, the detection rates in the control group ranged from 63–69% at low concentrations, 77–94% at medium concentrations, and 94–97% at high concentrations. In the C19-G, the corresponding ranges were 48–68%, 84–94%, and 94–100%, respectively. Detailed age-stratified detection rates are shown in Supplementary Table S1.

## Secondary outcomes: feasibility, comprehension, and retest reliability

### Feasibility and task comprehension

**Fig. 2:**
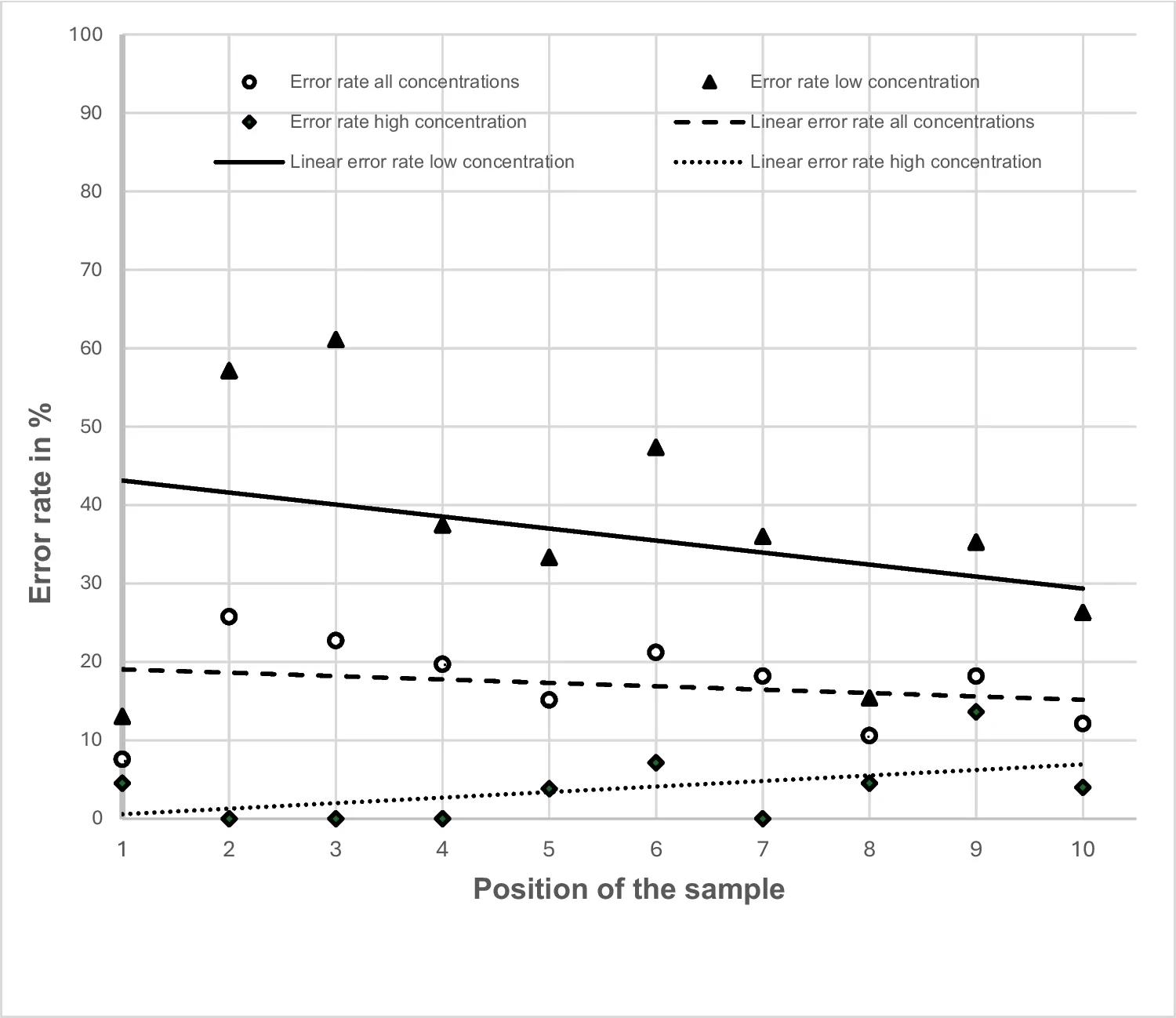
Error rates across sample positions. Mean percentages of incorrect classifications at each sample position (1–10), pooled across participants and irrespective of taste quality, shown for low-concentration stimuli (triangles), high-concentration stimuli (diamonds), and all concentrations combined (circles). The solid, dashed, and dotted lines indicate linear fits for low-concentration stimuli, all concentrations combined, and high-concentration stimuli, respectively

The dropout rate was 3%, indicating high feasibility (2/68 children). Task comprehension was also high: 59/66 (89.4%) correctly classified the water control (CG 32/35, 91.4%; C19-G 27/31, 87.1%), and four of seven water-control misclassifications occurred among three-year-olds (57.1%). The mean error rates across the ten sample positions remained stable (Fig. 2), suggesting no relevant fatigue effect.

Errors across the 10 samples were most frequent among the youngest children: three-year-olds had the highest error rate (43/170, 25.3%), compared with four-year-olds (19/150, 12.7%), five-year-olds (31/190, 16.3%), and six-year-olds (20/150, 13.3%).

### Retest reliability

**Fig. 3:**
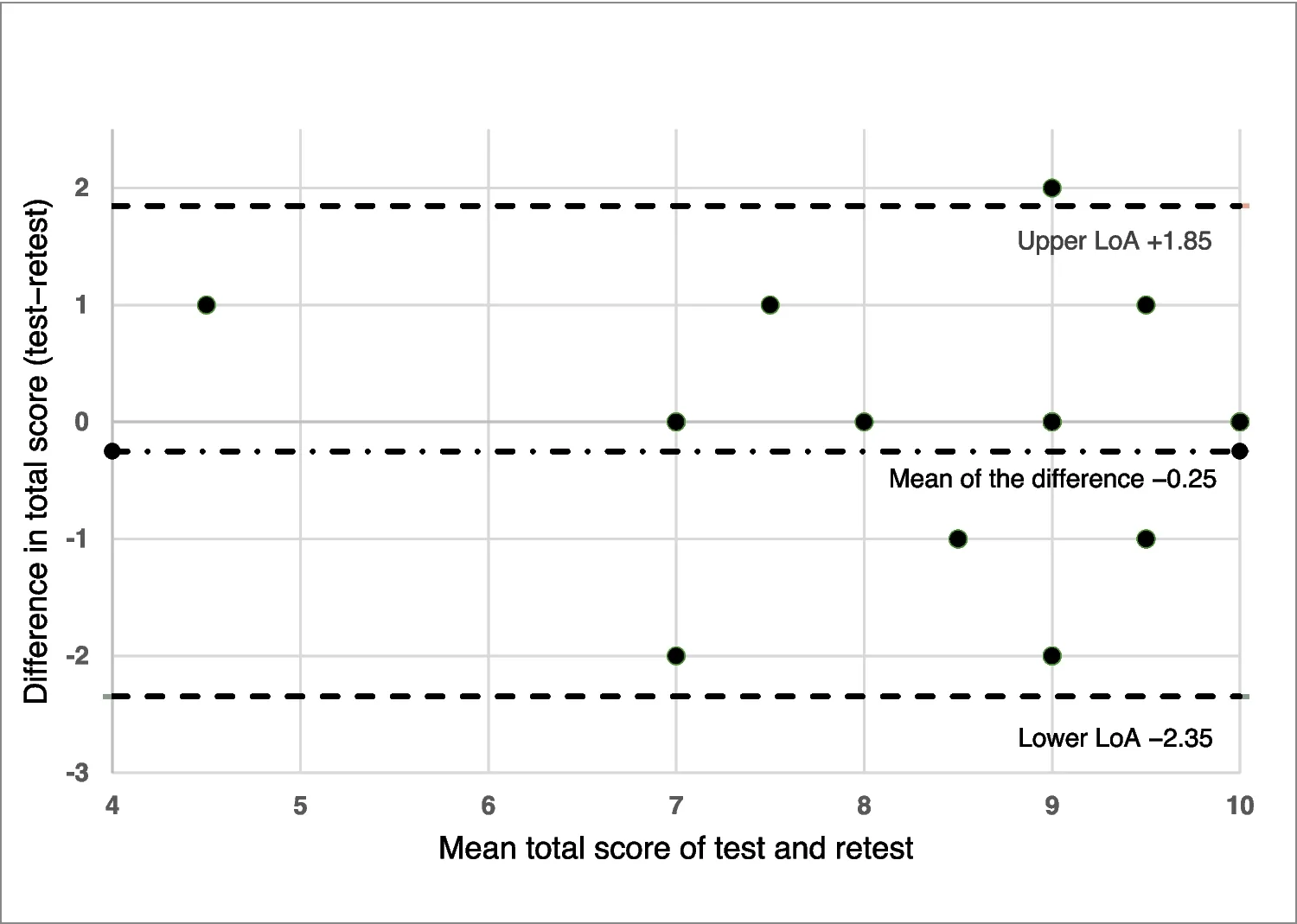
Bland–Altman agreement plot for the TasTY total score (test vs. retest). Individual test–retest differences in total scores (y-axis) are plotted against the mean of both measurements (x-axis). The dash-dotted line indicates the mean difference (bias =  − 0.25), and the dashed lines indicate the 95% limits of agreement (LoA; ± 1.96 SD)

All 20 retest participants completed both sessions. The mean total scores increased slightly from test to retest (8.40 ± 1.39 to 8.65 ± 1.53; p = 0.29), whereas the cumulative response time decreased (49.24 ± 13.35 s to 42.70 ± 16.36 s; p = 0.11). Across age strata, score changes showed no consistent age-dependent pattern, whereas response times decreased at retest (Table 3). The detection rates across all the stimuli remained stable. For the total score, the ICC was 0.73 (95% CI 0.44–0.89), with a pooled SEM of 0.76 points and an SRD of 2.11 points. Bland–Altman analysis revealed a small mean difference (bias) of − 0.25 points, with the 95% LoA ranging from − 2.35 to + 1.85 points, including one outlier (Fig. 3).

**Table 3:** Test–retest performance in the retest group (RG) by age

Measure	3 years (*n* = 5)	4 years (*n* = 6)	5 years (*n* = 4)	6 years (*n* = 5)	Overall (*n* = 20)
Test score	7.80 (6.0–9.2)	9.00 (8.3–9.7)	8.00 (7.0–9.0)	8.60 (8.0–9.2)	8.40 (7.7–9.1)
Response time, s	48.4 (36.4–63.4)	51.2 (38.4–69.2)	49.3 (42.2–56.3)	47.7 (40.2–55.8)	49.3 (44.1–54.8)
Retest score	7.60 (5.6–9.0)	9.17 (8.2–9.8)	9.25 (8.5–10.0)	8.60 (7.4–9.6)	8.65 (8.0–9.3)
Response time, s	47.6 (32.6–66.6)	37.1 (30.7–44.0)	44.3 (32.1–58.3)	43.1 (27.8–61.6)	42.7 (36.0–49.8)

Data are presented as means with bootstrapped 95% confidence intervals (CIs) for the TasTY total score (0–10) and cumulative response times at test and retest, by age group and overall. Cumulative response time was defined as the sum of the response times from ingestion to decision across all 10 samples, in seconds

In exploratory subscore analyses, test‒retest correlations were significant for the salty and control solutions, whereas Wilcoxon signed-rank tests revealed no significant test‒retest differences for the sweet, sour, salty, or control solutions. Detailed subscore results are shown in Supplementary Table S2.

## Discussion

We developed TasTY, a brief, child-adapted, objective taste test for preschoolers. However, even highly accurate tests have limited value if children are unable or unwilling to complete them. To address this challenge, TasTY balances test quality and feasibility in this challenging population. This balance is supported by high completion rates, good comprehension, test–retest reliability, and stable error rates.

Total taste scores did not differ between children with and without prior SARS-CoV-2 infection. Exploratory analyses suggested lower and more variable performance among three-year-olds than among older preschool children.

Previous pediatric studies investigated individual components of taste assessment, including detection thresholds [25, 27], sensitivity, preferences, and reliability [25, 27, 28, 33], as well as feasibility and child-adapted testing procedures [25, 27, 28]. These studies used different approaches, including the narrative setting of the Magic Water Test [28] and water-based forced-choice discrimination [27, 28, 40]. The unique contribution of TasTY is the combination of these previously separate elements in a double-blind, 10-sample design with randomized sample order and a standardized 0–10 score, providing a quantifiable outcome for direct group comparisons and longitudinal assessment. Feasibility was high, with a dropout rate of only 3%, despite younger children being particularly prone to dropout in previous pediatric taste studies [25, 27, 28]. In addition, 89.4% correctly identified the water control, supporting task comprehension.

Although scores did not differ across all four age groups, an exploratory Bonferroni-adjusted comparison indicated lower scores among three-year-olds than among children aged 4–6 years. This pattern is developmentally plausible and aligns with pediatric studies showing greater vulnerability of younger children to non-sensory influences, such as demands on attention, task comprehension, and working memory [28, 41, 42]. Accordingly, three-year-olds more often misclassified the water control, our predefined indicator of task comprehension. Each additional year of age corresponded to an estimated 0.30-point higher score, although nonsignificantly. Female sex also had a positive but nonsignificant coefficient, consistent with reports of better taste performance in female and older participants [18, 22, 41–43].

Prior SARS-CoV-2 infection was not associated with lower total taste scores, suggesting that no persistent quantitative taste reduction was detectable within TasTY’s sensitivity range. This should be interpreted in light of the upper-end score clustering, which limits discrimination. However, the simple test format enabled high participation. These results align with reports in children and adults that SARS-CoV-2-related taste disturbances became less frequent during later pandemic phases, particularly Omicron predominance, potentially reducing the likelihood of detectable postinfectious differences in taste sensitivity [8, 9, 13, 44]. Time since infection was likewise unrelated to total score, which was not significantly lower in the C19-G despite more frequent ENT-associated complaints. Isolated single-stimulus differences after stratification by age, taste quality, and concentration should be interpreted cautiously, because they arose in small subgroups and were not reflected in the overall score. Although TasTY was developed in connection with SARS-CoV-2, it may also be applicable to other conditions and treatments associated with quantitative taste dysfunction.

Total scores remained stable across sessions, and test–retest reliability was good and comparable to findings among older children [33]. Bland–Altman analysis showed minimal systematic bias and expected within-child variation, complementing reliability coefficients through visualization of agreement [37]. The pooled SEM of 0.76 points indicates expected within-child variation in repeated scores attributable to measurement error. Changes exceeding the SRD of 2.11 points, equivalent to at least 3 TasTY points, are more likely to reflect true change than measurement error. Shorter retest response times probably reflect habituation rather than altered taste performance. Together, the ICC, SEM, SRD, and Bland‒Altman analyses provide a more complete picture of reliability, covering relative ranking, single-measurement precision, and expected within-child variation; the SRD defines the minimum change likely to exceed measurement error [37, 38]. More broadly, objective taste assessment is relevant because taste disturbances may affect nutrition, growth, eating habits, and well-being [1–3]. TasTY moves beyond parental and subjective reports and may support longitudinal assessment of preschool children with taste-related conditions.

### Limitations and strengths

TasTY’s age-appropriate narrative and standardized, blinded, randomized procedure with an embedded water control supported motivation, objective assessment, task comprehension, and near-complete participation in this challenging preschool population; good test–retest reliability and stable error rates across sample positions further support practicality. Limitations include the small sample size, limiting statistical power and subgroup analyses; the retest subgroup was reduced by nonresponse and interim SARS-CoV-2 infections. Although concentrations were based on published pediatric threshold data, detection rates—particularly at medium and high concentrations—were higher than expected, resulting in upper-end score clustering, limited discrimination, and reduced sensitivity for mild quantitative taste deficits. Because TasTY included nine flavored samples and one water control, a tendency to classify samples as flavored may have inflated total scores and contributed to the ceiling effect. Infection status was determined retrospectively from parental reports and testing history; silent infections cannot be excluded, and the four-month postinfection window may have allowed recovery before assessment. Regional tap water likely improved familiarity and acceptability but may have limited comparability across settings. TasTY assessed only three of five basic taste qualities, reflecting a trade-off between comprehensiveness and feasibility, and could not capture qualitative disturbances such as parageusia. External validation against established psychophysical reference methods remains necessary.

## Conclusion

TasTY provides a feasible, age-adapted, objective measure of quantitative taste sensitivity in preschool children, moving beyond parental and subjective reports and supporting standardized group comparisons and longitudinal assessment. Beyond SARS-CoV-2, it may also be applicable to diseases and treatments associated with taste dysfunction. With further validation and normative reference data, TasTY may help close the current gap in objective taste assessment in this age group.

## Supplementary Information

Supplementary Material 1 (PDF 4.00 MB)

## Data Availability

The data supporting the findings of this study and additional study materials are available from the corresponding author upon reasonable request, subject to applicable ethical and data protection requirements.

## Abbreviations

2AFC: Two-alternative forced-choice
C19-G: Group of children with prior SARS-CoV-2 infection
CG: Control group
CI: Confidence interval
COVID-19: Coronavirus disease 2019
DIN ISO: German Institute for Standardization/International Organization for Standardization
ENT: Ear, nose, and throat
ICC: Intraclass correlation coefficient
IQR: Interquartile range
LoA: Limits of agreement
PCR: Polymerase chain reaction
ρ (rho): Spearman’s rank correlation coefficient
RG: Retest group
SARS-CoV-2: Severe acute respiratory syndrome coronavirus 2
SD: Standard deviation
SEM: Standard error of measurement
SPSS: Statistical Package for the Social Sciences
SRD: Smallest real difference
TasTY: Taste Test for Young Children

## Declarations

### Clinical trial registration

German Clinical Trials Register (DRKS), DRKS00030718, retrospectively registered on 12 December 2022 | https://drks.de/search/en/trial/DRKS00030718
