# Supplementary material for: TasTY: Development and evaluation of a taste test for quantifying taste sensitivity among preschool children—test–retest reliability, application, and comparison between children with and without prior SARS-CoV-2 infection
Source: Eur J Pediatr. 2026 Oct 19;185(11):858. doi: 10.1007/s00431-026-07534-w (PMC13677631; doi:10.1007/s00431-026-07534-w)
Supplement: Supplementary file 1 [file 431_2026_7534_MOESM1_ESM.pdf]

TasTY: Development and evaluation of a taste test for quantifying taste sensitivity among preschool children—test–retest reliability, application, and comparison between children with and without prior SARS-CoV-2 infection

Johannes Weißenfeld[1], Christoph Maier[1], Christine H. Meyer-Frießem[2], Anna Teresa Hoffmann[1], Thomas Lücke[1], Anne Schlegtendal[1]

Affiliations

1 University Children's Hospital, St. Josef Hospital, Ruhr University Bochum, Bochum, Germany

2 Department of Anaesthesiology, Intensive Care and Pain Medicine, KLW St. Paulus GmbH St. Marien Hospital Lünen, Germany; ORCID: 0000-0001-8743-2065

Journal: European Journal of Pediatrics

Corresponding author: Dr. med. Anne Schlegtendal
E-Mail: Anne.Schlegtendal@ruhr-uni-bochum.de

Clinical trial registration: German Clinical Trials Register (DRKS), TRN: DRKS00030718, retrospectively registered on 12 December 2022 | https://drks.de/search/en/trial/DRKS00030718

Supplementary Information

Supplementary Text S1 Dinosaur story to introduce the child to the test

"Today, you're going to need your super taste powers—because a little dinosaur needs your help! Once upon a time, a dinosaur mom had a baby who wasn't feeling well. To help the baby get better, she prepared a special magic potion and poured it into several cups. But oh no! While she was away, the dinosaur grandma came to visit and accidentally made a big mess in the kitchen, mixing up all the cups. Now no one knows which ones contain the magic potion and which are just plain water. Here's the tricky part: the potion looks just like water, so you can't tell just by looking—you have to taste it! That's because the magic potion tastes a little different from regular water, but only children can taste the difference. Grown-ups and dinosaurs can't tell at all. That's why the dinosaur mom needs you! You're the only one who can help find the right potion to make her baby feel better. To get you started, the dinosaur mom gives you one cup that she's absolutely sure has plain water. You can take a sip, so you know what regular water tastes like. After that, your mission begins—taste the other cups and try to find the magic potion!"

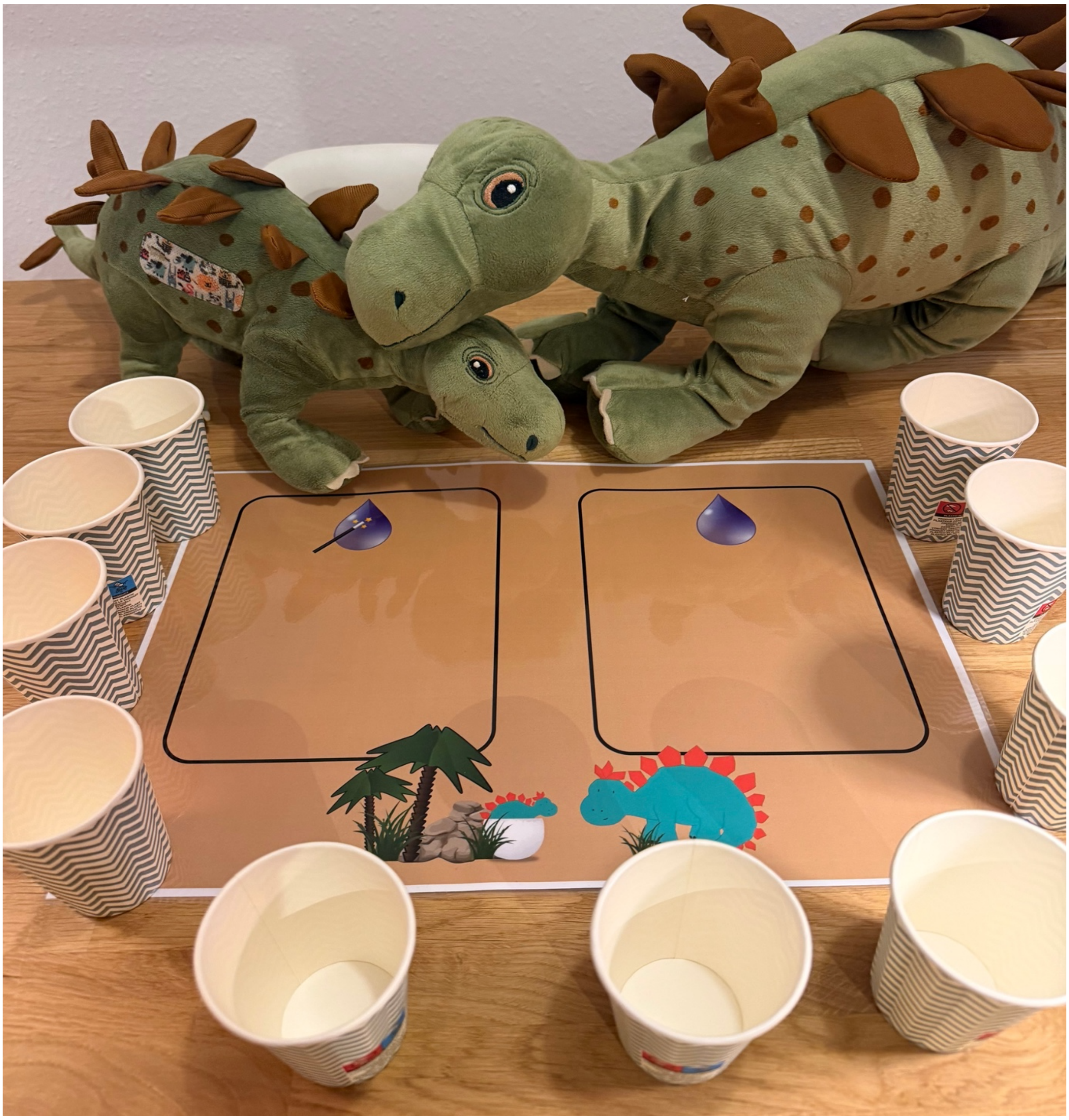

Supplementary Fig. S1 Test setup and playing board

Reconstructed test setup with 10 coded samples in paper cups, two stuffed dinosaurs to encourage participation, and a playing board with two marked fields for the two-alternative forced-choice (2AFC) task. The field on the left represents flavored samples ("magic potion") and is marked by a magic wand and stars within a water droplet; the field on the right represents unflavored water.

Note to Fig. S1: The test setup was reconstructed to closely match the original and photographed after completion of the study to avoid uncertainty regarding publication rights for the original illustrations. The board's layout and response options were retained, while the decorative illustrations were replaced with Openclipart images under CC0 1.0. This replacement concerns only the published illustration and does not affect the study data, analyses, results or conclusions. Sources and links are listed at the end of this supplementary file.

Supplementary Table S1 Detection rates by taste quality, concentration, and age group in the control group (CG) and children with prior SARS-CoV-2 infection (C19-G)

Age	Taste	CG low	C19-G low	CG medium	C19-G medium	CG high	C19-G high
Overall	Sweet	24/35 (69%)	15/31 (48%)	27/35 (77%)	26/31 (84%)	34/35 (97%)	30/31 (97%)
3 years	Sweet	7/11 (64%)	4/6 (67%)	6/11 (55%)	5/6 (83%)	10/11 (91%)	5/6 (83%)
4 years	Sweet	5/6 (83%)	3/9 (33%)	6/6 (100%)	8/9 (89%)	6/6 (100%)	9/9 (100%)
5 years	Sweet	5/10 (50%)	6/9 (67%)	8/10 (80%)	9/9 (100%)	10/10 (100%)	9/9 (100%)
6 years	Sweet	7/8 (88%)	***2/7 (29%)******	7/8 (88%)	4/7 (57%)	8/8 (100%)	7/7 (100%)
Overall	Sour	22/35 (63%)	19/31 (61%)	33/35 (94%)	27/31 (87%)	33/35 (94%)	29/31 (94%)
3 years	Sour	5/11 (45%)	4/6 (67%)	9/11 (82%)	6/6 (100%)	9/11 (82%)	5/6 (83%)
4 years	Sour	4/6 (67%)	5/9 (56%)	6/6 (100%)	9/9 (100%)	6/6 (100%)	9/9 (100%)
5 years	Sour	7/10 (70%)	5/9 (56%)	10/10 (100%)	***5/9 (56%)******	10/10 (100%)	8/9 (89%)
6 years	Sour	6/8 (75%)	5/7 (71%)	8/8 (100%)	7/7 (100%)	8/8 (100%)	7/7 (100%)
Overall	Salty	24/35 (69%)	21/31 (68%)	31/35 (89%)	29/31 (94%)	33/35 (94%)	31/31 (100%)
3 years	Salty	8/11 (73%)	2/6 (33%)	10/11 (91%)	4/6 (67%)	9/11 (82%)	6/6 (100%)
4 years	Salty	4/6 (67%)	8/9 (89%)	5/6 (83%)	9/9 (100%)	6/6 (100%)	9/9 (100%)
5 years	Salty	7/10 (70%)	6/9 (67%)	8/10 (80%)	9/9 (100%)	10/10 (100%)	9/9 (100%)
6 years	Salty	5/8 (63%)	5/7 (71%)	8/8 (100%)	7/7 (100%)	8/8 (100%)	7/7 (100%)

Values are n/N (%), where n is the number of correct detections and N is the number tested within each stratum at low, medium, and high concentrations. * Nominal $p < 0.05$ for the CG versus the C19-G within the respective stratum (unadjusted χ^2 test).

Supplementary Fig. S2 Distribution of TasTY total scores by age group

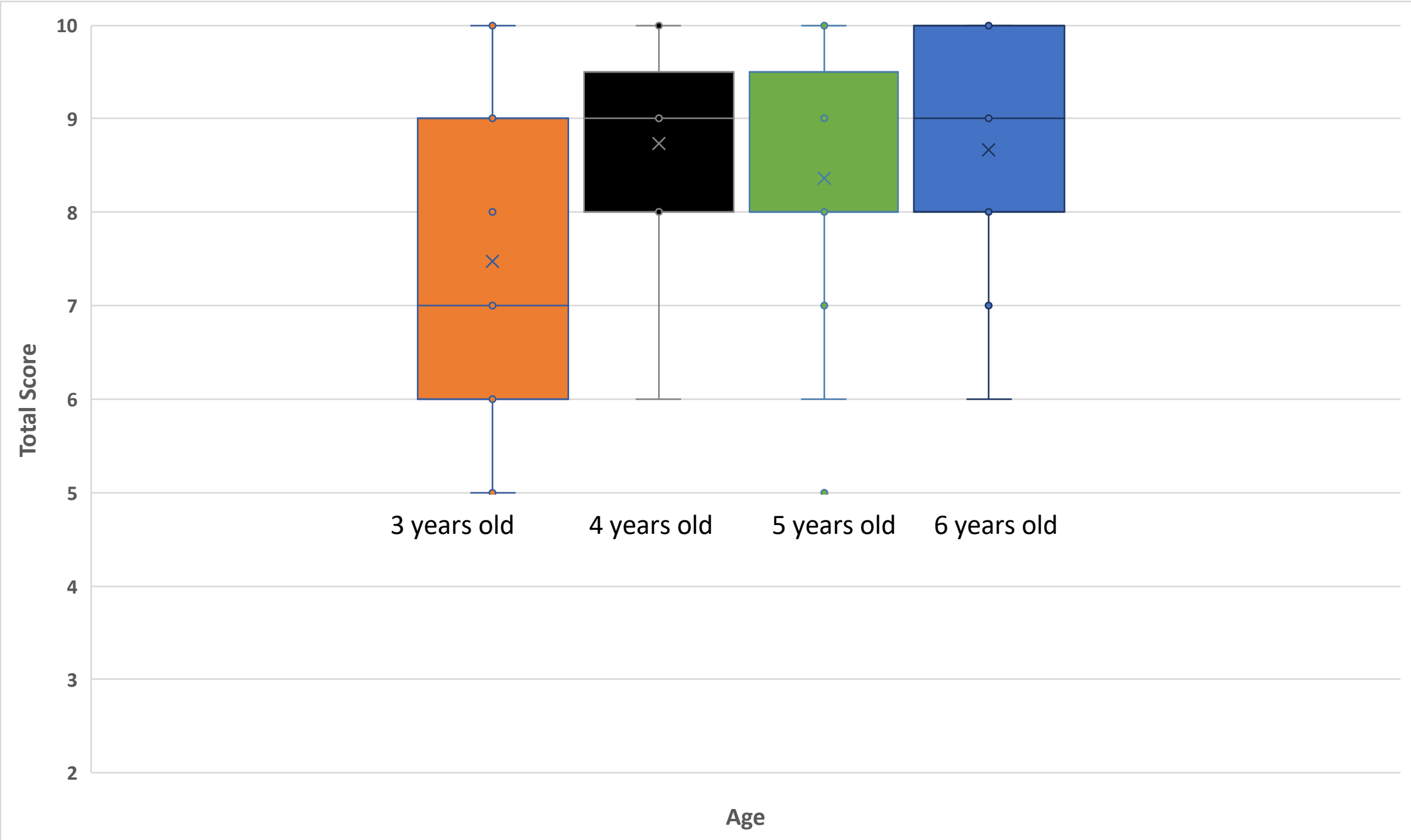

Box plots show the distribution of TasTY total scores (0–10) among children aged 3, 4, 5, and 6 years, pooled across the control group (CG) and children with prior SARS-CoV-2 infection (C19-G). Boxes represent the interquartile range (IQR), the horizontal lines within each box indicate the medians, whiskers extend to the most extreme non-outlier values, crosses indicate means, and dots represent individual observations, including outliers.

Supplementary Table S2 Test–retest subscore results by taste quality in the retest group (RG)

	N	Test score (SD)	Retest score (SD)	Spearman's ρ	p, correlation	Wilcoxon Z	p, Wilcoxon
Sweet	20	2.45 (0.76)	2.80 (0.41)	0.33	0.150	−1.93	0.053
Sour	20	2.70 (0.47)	2.50 (0.69)	0.35	0.133	−1.27	0.206
Salty	20	2.40 (0.88)	2.50 (1.00)	**0.78***	**< 0.001**	−0.63	0.527
Control solution	20	0.85 (0.37)	0.85 (0.37)	**0.61***	**0.004**	0.00	1.000

Data are presented as means (SDs) for test and retest subscores (sweet, sour, and salty: 0–3; control solution: 0–1), together with Spearman's ρ (two-tailed) and Wilcoxon signed-rank test results (Z and p). *p < 0.05 for Spearman's correlation.

Image credits for Supplementary Fig. S1

Dinosaurs: https://openclipart.org/334466

Water droplet: https://openclipart.org/4063

Magic wand: https://openclipart.org/83863

Palm trees: https://openclipart.org/3914

Rocks: https://openclipart.org/208778

Grass: https://openclipart.org/167346

Eggshell: https://openclipart.org/8016

CC0 1.0: https://creativecommons.org/publicdomain/zero/1.0/
